# Automatic RadLex coding of Chinese structured radiology reports based on text similarity ensemble

**DOI:** 10.1186/s12911-021-01604-9

**Published:** 2021-11-16

**Authors:** Yani Chen, Shan Nan, Qi Tian, Hailing Cai, Huilong Duan, Xudong Lu

**Affiliations:** 1 College of Biomedical Engineering and Instrument Science, Zhejiang University, Zheda Road, Hangzhou, 310027 China; 2 Key Laboratory for Biomedical Engineering, Ministry of Education, Hangzhou, China; 3 School of Industrial Engineering, Eindhoven University of Technology, Eindhoven, The Netherlands; 4 School of Biomedical Engineering, Hainan University, Haikou, China

**Keywords:** Automatic coding, Hybrid translation, Text similarity ensemble, Standardized radiology reports

## Abstract

**Background:**

Standardized coding of plays an important role in radiology reports’ secondary use such as data analytics, data-driven decision support, and personalized medicine. RadLex, a standard radiological lexicon, can reduce subjective variability and improve clarity in radiology reports. RadLex coding of radiology reports is widely used in many countries, but translation and localization of RadLex in China are far from being established. Although automatic RadLex coding is a common way for non-standard radiology reports, the high-accuracy cross-language RadLex coding is hardly achieved due to the limitation of up-to-date auto-translation and text similarity algorithms and still requires further research.

**Methods:**

We present an effective approach that combines a hybrid translation and a Multilayer Perceptron weighting text similarity ensemble algorithm for automatic RadLex coding of Chinese structured radiology reports. Firstly, a hybrid way to integrate Google neural machine translation and dictionary translation helps to optimize the translation of Chinese radiology phrases to English. The dictionary is made up of 21,863 Chinese–English radiological term pairs extracted from several free medical dictionaries. Secondly, four typical text similarity algorithms are introduced, which are Levenshtein distance, Jaccard similarity coefficient, Word2vec Continuous bag-of-words model, and WordNet Wup similarity algorithms. Lastly, the Multilayer Perceptron model has been used to synthesize the contextual, lexical, character and syntactical information of four text similarity algorithms to promote precision, in which four similarity scores of two terms are taken as input and the output presents whether the two terms are synonyms.

**Results:**

The results show the effectiveness of the approach with an F1-score of 90.15%, a precision of 91.78% and a recall of 88.59%. The hybrid translation algorithm has no negative effect on the final coding, F1-score has increased by 21.44% and 8.12% compared with the GNMT algorithm and dictionary translation. Compared with the single similarity, the result of the MLP weighting similarity algorithm is satisfactory that has a 4.48% increase compared with the best single similarity algorithm, WordNet Wup.

**Conclusions:**

The paper proposed an innovative automatic cross-language RadLex coding approach to solve the standardization of Chinese structured radiology reports, that can be taken as a reference to automatic cross-language coding.

## Background

Radiology examination plays an increasingly important role in the diagnosis and treatment of disease. After a radiology imaging procedure, radiologists will read the images and make a report summarizing the findings and impressions, which can be used as references for further decision processes of physicians. Due to the differences in education and experiences, radiologists make radiology reports with different styles of expression and use various phrases to describe the same condition. The consistent coding of radiology reports is a common requirement for anyone who wants to re-use radiology reports for further research [1]. Standardized coding of radiology reports provides many benefits: reducing ambiguity, improving the communication between radiologists and clinicians, and increasing the ability to mine data for research which results in speedier diagnosis and treatment [2]. However, it remains an ongoing challenge due to non-standard terms in the radiology reports.

Beginning in 2005, the Radiological Society of North America convened radiology experts to create RadLex [3], a rich and structured radiology-specific lexicon. With proven accuracy and accolades from the radiologist, RadLex provides a standard set of radiological terms with codes that improve clarity in radiology reports. Most of the RadLex coding tasks are done manually at present, the coding process involves much clinical knowledge and the coding person needs to look up code by knowing the exact location or by browsing a large list of standard terms [4], the inefficiency of manual coding and the lack of coding professionals make it hard to code even one radiology report. Speeding up and facilitating the tedious process of manual coding remain a priority [5]. Compared with traditional manual coding, automatic coding can speed up 25% to 43% [6], which is widely recognized as essential for standardized coding of medical data [7].

There have been many studies on automated or semi-automated coding of English radiology reports to improve the productivity of coders. For example, Farkas et al. constructed rule-based coding systems for radiology reports [8]. Emily et al. developed a validated natural language processing algorithm for brain radiology reports [9]. Stefano et al. applied hierarchical supervised learning technology to the problem of assigning codes to radiology reports [10]. However, these methods are all based on an alphabetic system of English and not suitable for Chinese radiology reports using a logographic system without an alphabet. In logographic systems, symbols represent the words themselves—words are not made up of various letters as in alphabetic systems.

In recent years, there are some studies on Chinese automatic medical coding. Ning et al. developed a hierarchical approach to automatically encoded Chinese diagnoses with ICD-10 codes through semantic similarity estimation [11]. Chen et al. presented an approach based on the Longest Common Subsequence and semantic similarity for automatic Chinese diagnoses coding [12]. But none of these studies were related to RadLex coding in Chinese.

The lack of a specialized radiology standard lexicon is a big challenge for Chinese radiology researchers. Although RadLex coding of radiology reports is widely used in many countries, it is difficult to implement in China since translation and localization of RadLex are still far from been established. To translate the RadLex lexicon, not only a lot of manpower and resources are needed, but the various synonyms for one RadLex term will be lost after the translation. The central problem of automatic coding of Chinese radiology reports is how to map Chinese radiological phrases with codes from the English radiology standard lexicon. The automatic cross-language RadLex coding can quickly alleviate the current less standardized radiology reports, which is a practical method without the necessity of translating the RadLex lexicon. So cross-language automatic coding will become the most promising direction of Chinese radiology reports standardization research.

The remainder of the paper is organized as follows. Section 2 summaries methods. Section 3 describes the performance of the hybrid translation algorithm and the Multilayer Perceptron weighting text similarity ensemble algorithm compared with other translation and text similarity algorithms. Section 4 discusses the results and suggests possible directions for future work. Section 5 concludes the paper.

## Methods

**Fig. 1:**
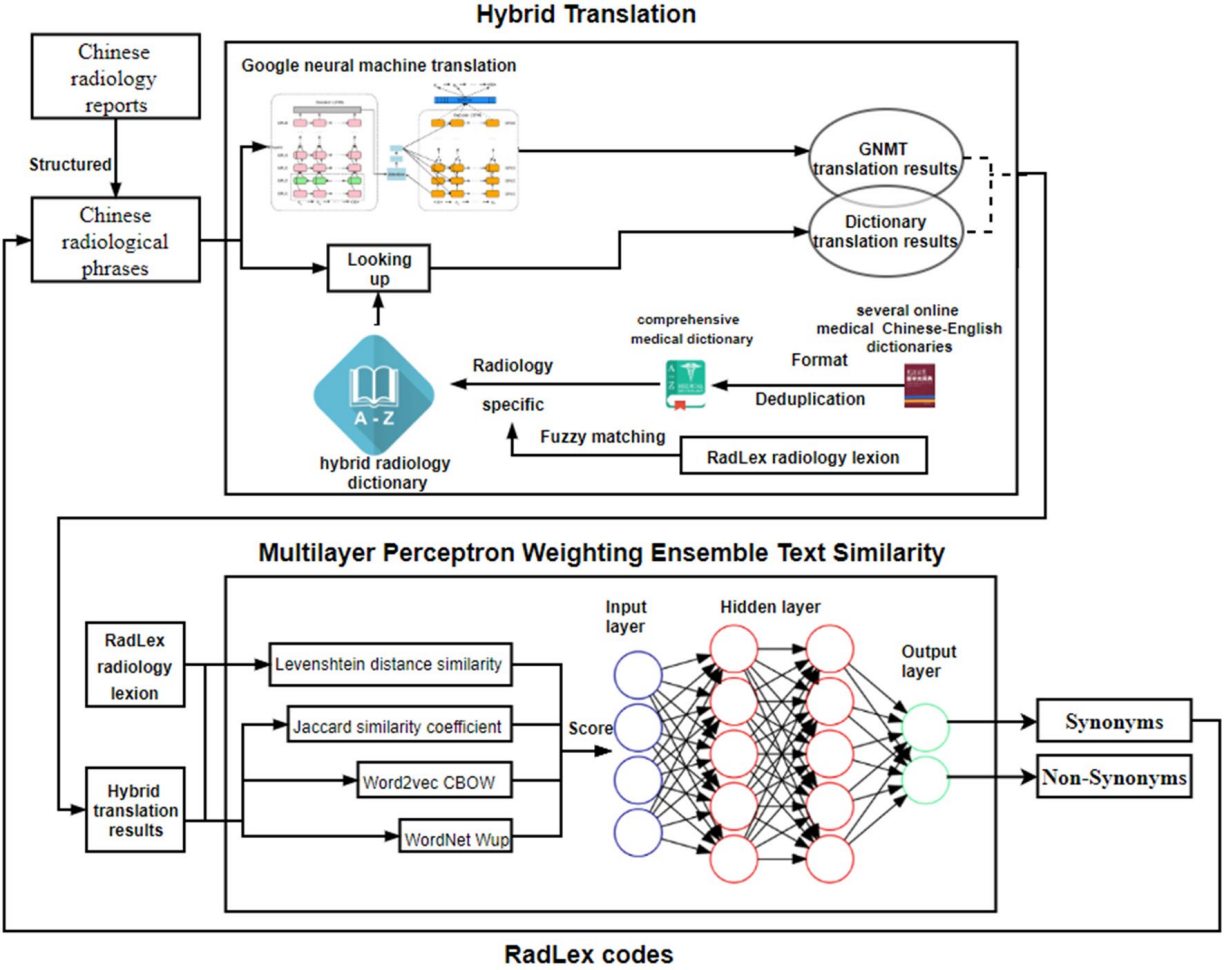
The framework of the automated RadLex coding approach

There are two steps in developing an effective automatic RadLex coding approach: hybrid translation and Multilayer Perceptron (MLP) weighting text similarity ensemble algorithm. The framework of this approach is shown in Fig. 1. In the part of the hybrid translation, the hybrid radiology dictionary is constructed by integrating several available medical Chinese–English dictionaries and fuzzy matching with RadLex. The union of the dictionary translation results and Google translation results is used in the subsequent similarity calculation. Afterward, in the part of MLP weighting text similarity ensemble algorithm, the results of four typical text similarity algorithms, which are Levenshtein distance similarity, Jaccard similarity coefficient, Word2vec Continuous bag-of-words model (CBOW), WordNet Wup, are used as the input of MLP to determine whether the two terms are synonyms and whether Chinese radiological phrases can be encoded with RadLex codes.

### Structured radiology report Chinese-to-English translation

Auto-translation has made leap-forward progress, especially Google neural machine translation, but available translation algorithms can work only with text in general domain. Many professional radiological phrases cannot be accurately translated by existing available translation algorithms.

**Fig. 2:**
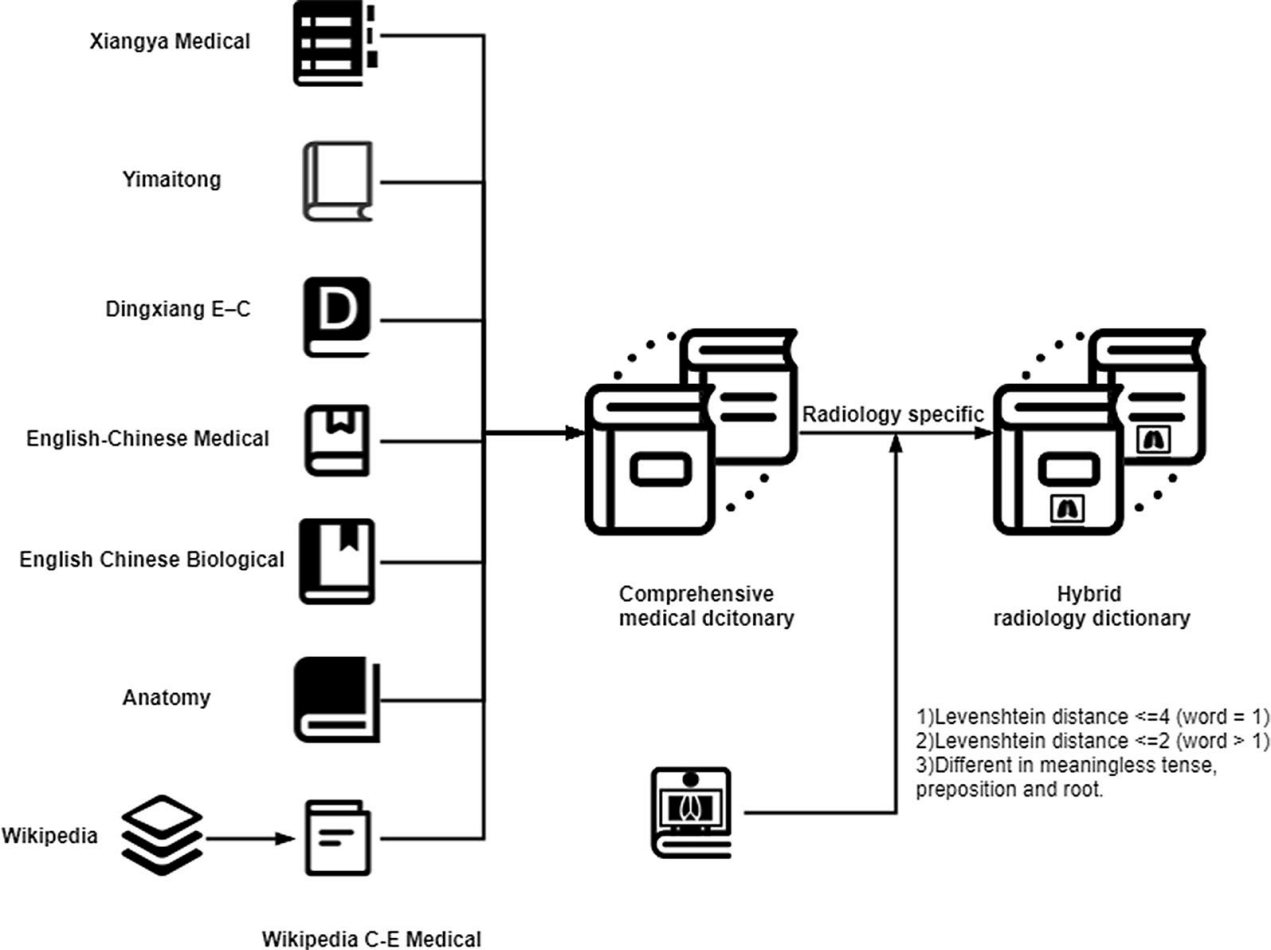
The construction of hybrid radiology dictionary

For better Chinese-to-English translation of radiological phrases, several online medical Chinese–English dictionaries were used to construct a radiology dictionary shown as Fig. 2, including Xiangya Medical Dictionary (dict.biomart.cn), Yimaitong (meddic.medlive.cn/index.do), Dingxiang E–C Dictionary (mcd8.com), English–Chinese medical dictionary (esaurus.org), English Chinese biological dictionary (cmi.hku.hk/Ref/Glossary/Bio/a.htm), anatomy dictionary (dict.bioon.com/elite.asp) and Wikipedia C–E medical dictionary that extracted Chinese and English expressions from the medical entries in Wikipedia. A comprehensive English–Chinese medical dictionary was then constructed after combining and deduplicating all the above dictionaries [13]. As a result, the comprehensive medical dictionary was made up of 731,175 medical terms, as shown in Table 1.

**Table 1:** Chinese–English medical dictionaries

Chinese–English medical dictionaries	Number of term pairs
Xiangya Medical Dictionary	404,334
Yimaitong Dictionary	174,582
Dingxiang E–C Dictionary	627,947
English–Chinese Medical Dictionary	2156
English Chinese Biological Dictionary	5841
Anatomy Dictionary	1189
Wikipedia C–E Medical Dictionary	4446
Deduplicate total	731,175

The comprehensive medical dictionary has a desirable coverage but becomes a disaster to the subsequent quick-look-up translation. The extraction of radiology-specific terms is of great help to promote the efficiency of dictionary translation. Most radiology-specific terms from the above comprehensive medical dictionary are written completely identical or slightly different from terms in RadLex. The differences in the expressions of English synonyms are mainly intense, preposition, and part of speech [14]. If the terms in the dictionary are different in meaningless tense, preposition, and suffix/postfix with ones in RadLex, they will be regarded as fuzzy matched. There are many fuzzy matching methods to locate radiology-specific terms, among which the Levenshtein Distance is the most widely used through measuring term similarity in terms of the number of operations necessary to convert one string into another. If the Levenshtein distance is less than 4 between the terms in the dictionary and RadLex, except for terms with only one word, it will be considered that the terms in the dictionary can be fuzzy matched with the terms in RadLex. After fuzzy matching, a hybrid radiology dictionary was achieved and made up of 21,863 radiology terms.

Although the English term could be precisely translated through looking-up the dictionary, it is limited by the coverage. If there is no such radiological term in the dictionary, the translation will have no result and has a negative impact on subsequent coding. Compared to the dictionary translation, Google neural machine translation can produce fluent translation without the problem of limited coverage but with a lack of accuracy. Some rules were used to search the exact match of Chinese terms in the Chinese–English radiology dictionary. If the matching results cannot be found in the dictionary, the Google translation was used to translate. Online Chinese–English dictionaries were integrated as much as possible to achieve the highest possible translation quality. A hybrid way to integrate Google neural machine translation and dictionary translation helped to optimize translation results for radiological terms, where the results of the two methods were united. To be more specific, Google neural machine translation and dictionary translation were performed in parallel. If the results of the two are the same or only the result of the former method can be achieved, that the result will be taken. If the results of the two are partly the same or completely different, they will be kept in the union set and be both used for subsequent similarity calculations.

### Text similarity algorithms

Text similarity algorithms can be roughly classified into three classes, String-based, Corpus-based, and Knowledge-based similarities [15]. String-Based similarity algorithms measure the common information two texts shared [16, 17] and can be further classified into two main types, character-based and word-based. Corpus-Based similarity algorithms use the distribution of words within a corpus to represent the semantic similarity between two texts [18]. Knowledge-Based similarity algorithms are semantic similarity measures determined by the path of words in knowledge sources such as dictionaries, taxonomies, and semantic networks [19]. The typical algorithm in each category and the ensemble algorithm will be proposed as follows.

Four typical text similarity algorithms.

- Levenshtein distance similarity algorithm

The Levenshtein distance similarity algorithm is the most basic Character-based similarity algorithms. The Levenshtein distance between two words is the minimum number of single-character edits (insertions, deletions or substitutions) required to change one word into the other [20].

Levenshtein distance similarity algorithm is simple and easy, but the meaning of an individual character is usually ambiguous and one-sided, so the Levenshtein distance similarity algorithm can only represent the glyphs information and is only suitable in cases of short text. The glyph of many radiology terms is similar, but they have completely different meanings, such as “posterior zone of superior part proper of body of right scapula” and “posterior zone of superior part proper of body of left scapula”.

- Jaccard similarity coefficient algorithm

Comparing with the Character-Based algorithms, the Word-Based algorithms are more suitable for longer text, but will usually discard the order and location information and has a weakness for short text. Jaccard similarity coefficient is a typical algorithm of Word-based similarity algorithms. The Jaccard similarity coefficient is defined as the size of the intersection divided by the size of the union [21].

Jaccard similarity coefficient algorithm is efficient and widely used, but the similarity results are greatly affected by the number of words in the text. For example, the term “nodules” and the term “tuberosity” (RID39357) have relatively a low Jaccard similarity coefficient, but they are obviously a pair of synonyms.

- Word2vec CBOW algorithm

Character-based and Word-based similarity algorithms are short of semantic information, and corpus-based similarity algorithms use co-occurrence information to measure the semantic similarity of texts. Word2vec is a representative corpus-based similarity algorithm that can learn the vector representations of words in the high-dimensional vector space and calculate the similarity between texts [22]. Continuous bag-of-words (CBOW) and continuous skip-gram model are two architectures that can be leveraged by Word2Vec to create word embedding. In the CBOW model, the distributed representations of context are combined to predict the target word in the middle. The Skip-gram model reverses the use of target and context [23]. Compared to skip-gram, CBOW is more suitable for radiology reports coding since it is several times faster in the process of training and slightly better accuracy for the frequent words [22].

**Fig. 3:**
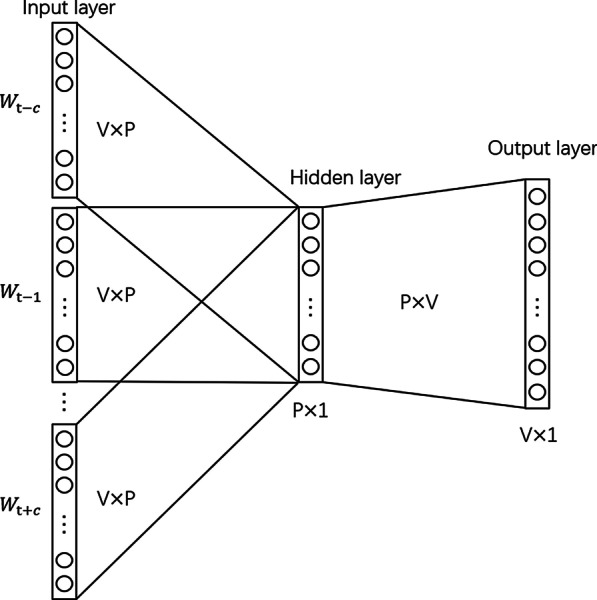
The structure of the CBOW model

In the process of CBOW, three layers were used and shown in Fig. 3. The input layer corresponded to the context. The hidden layer corresponded to the projection of each word from the input layer into the weight matrix which was projected into the output layer. The final step of the model was the comparison between its output and the word itself to correct its representation based on the backpropagation of the error gradient. Thus, the purpose of the CBOW neural network is to maximize the following [24]:

$$\frac{1}{V}\sum_{t=1}^{V}\mathrm{log }p\left({w}_{t}|{m}_{t-c}\dots {m}_{t+c}\right)$$

where *V* corresponds to vocabulary size, *c* corresponds to the window size of each word.

Word2Vec CBOW similarity algorithm needed a corpus to train the model, that collected from Wikipedia, 210 radiology report templates from RNSA, and the texts from imaging and nuclear medicine of MedAca. The corpus-based similarity algorithms inevitably encounter the problem of out-of-vocabulary terms with the limitation of the corpus. The out-of-vocabulary terms are taken as unknown words(UNK) that have a negative impact on coding results.

- WordNet Wup similarity algorithm

The knowledge-based similarity algorithms quantify the degree to which two words are semantically related using information from semantic networks. WordNet is one of the most popular and abundant semantic networks that contains semantic relations among words [25], Version 2.1 of WordNet is used in this study. There are several WordNet-based similarity algorithms, Wu and Palmer (Wup) similarity algorithm is an appropriate choice based on observed performance in other language processing applications and relatively low computational efficiency.

The Wup similarity algorithm takes into account the position of concepts in the taxonomy relative to the position of the Least Common Subsume (LCS) [26]. The LCS of two nodes *a* and *b* is the deepest node that has both *a* and *b* as descendants. To calculate the WordNet Wup similarity between two words *a* and *b*, ${N}_{a,LCS}$ is the number of nodes from *a* to $LCS$, and ${N}_{b,LCS}$ is the number of nodes from *b* to $LCS$. ${N}_{LCS}$ is the number of nodes from $LCS$ to the root node. ${Sim}_{wup}\left(a\text{,}\mathrm{b}\right)$ clearly indicates the WordNet Wup similarity of *a* and *b* as follows.

$${Sim}_{wup}\left(a\text{,}\mathrm{b}\right)=\frac{2\times {N}_{LCS}}{{N}_{a,LCS}+{N}_{b,LCS}}$$

The Wup similarity may give inaccurate coding results because two concepts in the same hierarchy may show a lower similarity than two concepts belonging to different hierarchies [27].

### Text similarity ensemble algorithm

Each of the above algorithms is based on a single feature and has some limitations. The text similarity ensemble algorithm combines multiple contextual, lexical, and syntactical features to achieve better results [28–30].

Linear weighting is a generalized method of ensemble text similarity, but there is no consensus on how to define proper weights. Besides, radiological terms vary in length and style, linear weighting lacks the flexibility to adequately address many radiological terms synonyms recognition problems. Using the neural networks in machine learning can dynamically and automatically assign the weight to overcome the limitations of linear weighting.

Neural networks, with the remarkable ability to derive information from complicated data, work well in prediction with clear inputs. There are many complex and effective neural network models for the classification problem, but the score of text similarity has no clear criterion and meaning. Multilayer Perceptron (MLP) is one of the most popular neural network models due to its clear architecture and comparably simple algorithm of extracting information. MLP is used for basic operations like algorithm weighting and data analytics [31], consisting of at least three layers: an input layer, at least one hidden layer, and an output layer. Except for the nodes of the input layer, each node is a neuron that uses a nonlinear activation function where every layer is a fully connected layer.

**Fig. 4:**
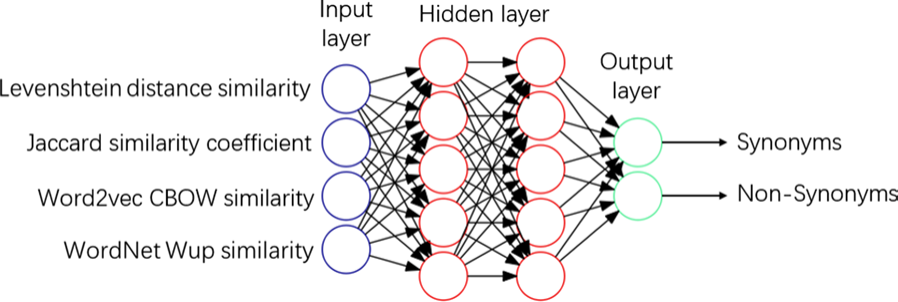
The structure of the MLP model

Traditionally there are various heuristics for choosing the number of hidden layers and nodes. The theory focused on semiparametric inference and gave a theoretically grounded starting point for choosing the architecture. Increasing the number of hidden layers generates a more robust multilayer perceptron but costs more time in processing. Two hidden layers with five neurons are a good choice [32]. An MLP model with two hidden layers was designed based on the above theories to integrate four text similarity algorithms (illustrated in Fig. 4).

Once the architecture of MLP has been determined, the connection weights must be computed through a training procedure based on the training patterns and the desired output. The backpropagation algorithm (BP) is one of the simplest and most general methods for the supervised training of MLP [33]. The text similarity scores from the above four similarity algorithms were used as the inputs of the MLP model. They were pushed forward through the dot product with the corresponding weights that existed between the input layer and the hidden layer, then yielded a value at the hidden layer. MLP utilized activation functions, rectified linear units (ReLU), to calculate output at the hidden layer and pushed it to the next layer by taking the dot product with the corresponding weights. The above steps were repeated until the output layer reached. If the desired output cannot be achieved in the output layer, then the error will be backpropagated. The connection weights were adjusted dynamically accordingly and the backpropagation stopped when the error of the output met the requirement. After training, the MLP model can be used to integrate the four similarity algorithms.

### Dataset preparing

6000 structured chest CT reports from a hospital in China between July 2016 and February 2017 were used as the dataset for the experiment. A total of 4046 radiology-specific phrases were extracted through an entity recognition method based on character embedding, Iterated Dilated Convolutional Neural Networks (IDCNN) and Conditional Random Fields (CRF) after de-duplication [34]. The manually annotated codes were assigned by two professional clinical coders from the RadLex after reading the phrases. The 3778 times consensus of two coders was reached to arrive at the gold standard for each coding. 70% of these coded phrases were employed for training and tuning the MLP model, while 30% rest were used to evaluate the performance of the trained model.

### Performance evaluation criteria

The precision, recall and F1-score are defined by the following formulas to evaluate the performance of the automatic RadLex coding algorithm.

True Positive (TP): Chinese radiological phrase with correct RadLex code.

False Negative (FN): Chinese radiological phrase with wrong RadLex code.

False Positive (FP): No-match Chinese radiological phrase that is recorded as matched.

$$\begin{aligned}\mathrm{precision}&=\frac{TP}{TP+FP}\\ \mathrm{recall}&=\frac{TP}{TP+FN}\\ {\hbox{F1-score}}&=\frac{2*\mathrm{precision}*\mathrm{recall}}{\mathrm{precision}+\mathrm{ recall}}\end{aligned}$$

## Results

### Evaluation of Chinese-to-English translation

It is very difficult to measure the translation of radiology reports directly since the golden standard of translation can hardly be achieved. Therefore, the final coding results were taken as the evaluation metric of translation quality when using the same MLP weighting text similarity ensemble algorithm. The translation evaluation carried out a four-way side-by-side (SxS) evaluation. The results were from (1) Google translation, (2) Dictionary translation, (3) Hybrid translation integrated Google neural machine translation and dictionary mapping, and (4) Translations by humans with expertise in Chinese to English. As shown in Table 2, hybrid translation is the next best to human translation and outperforms the Google translation and Dictionary translation respectively. Dictionary translation has high precision and low recall, whereas Google translation is the opposite. Hybrid translation united the results of them, slightly reduced the efficiency but greatly improved precision and recall. Automatic translation still has many limitations and deficiencies and cannot replace human translation, but reduce the cost and delivery time.

**Table 2:** Translation evaluation based on final coding results

Method	Precision (%)	Recall (%)	F1-score (%)
Google translation + MLP weighting text similarity ensemble algorithm	59.33	81.61	68.71
Dictionary translation + MLP weighting text similarity ensemble algorithm	87.42	77.27	82.03
Hybrid translation + MLP weighting text similarity ensemble algorithm	91.78	88.59	90.15
Human translation + MLP weighting text similarity ensemble algorithm	97.26	90.37	93.69

### Evaluation of automatic RadLex coding based on text similarity

The precision, recall, and F1-score evaluation of the four similarity algorithms and multiple combinations of four similarity algorithms were clearly compared under the same hybrid translation shown in Table 3. The MLP weighing algorithm outperforms the other four similarity algorithms in terms of precision, recall, and F1-score. The MLP weighing algorithm has the best performance with precision, recall, and F1-score of 91.78%, 88.59%, and 90.15%. The WordNet Wup similarity algorithm is next to the MLP weighing algorithm with precision, recall, and F1-score of 86.78%, 84.59%, and 85.67%. The Word2vec CBOW similarity algorithm is a little weaker than the WordNet Wup similarity algorithm with precision, recall, and F1-score of 81.75%, 81.16%, and 84.18%. The Jaccard and Levenshtein distance similarity algorithms have relatively poor performances. The MLP weighing algorithm has a 4.48% F1-score increase compared with the WordNet Wup method, has a 5.97% F1-score increase compared with the Word2vec CBOW method, has a 30.87% F1-score increase compared with the Levenshtein distance method, and has a 32.1% F1-score increase compared with the Jaccard method.

**Table 3:** Six-way side-by-side automatic coding evaluation

Method	Precision (%)	Recall (%)	F1-score (%)
Levenshtein distance	70.91	50.93	59.28
Jaccard	56.64	53.28	58.05
Word2vec CBOW	81.75	81.16	84.18
WordNet Wup	86.78	84.59	85.67
Levenshtein distance + Jaccard	66.90	50.85	57.78
Levenshtein distance + Word2vec CBOW	72.61	64.18	68.14
Levenshtein distance + WordNet Wup	78.52	67.38	72.52
Jaccard + Word2vec CBOW	68.29	66.47	67.37
Jaccard + WordNet Wup	70.22	67.72	68.95
Word2vec CBOW + WordNet Wup	83.95	82.41	83.17
Levenshtein distance + Jaccard + Word2vec CBOW	71.81	62.93	67.08
Levenshtein distance + Jaccard + WordNet Wup	73.17	71.43	72.29
Levenshtein distance + Word2vec CBOW + WordNet Wup	84.21	77.49	80.71
Jaccard + Word2vec CBOW + WordNet Wup	72.83	73.92	73.37
MLP weighing	91.78	88.59	90.15

Among these similarity algorithms, the String-Based similarity algorithms have relatively lower performance because the synonyms may have quite different glyphs, such as “cranium” and “skull”. The recognition of this type of synonyms can only be achieved through the semantic-based similarity algorithm. But semantic-based similarity relies on manpower and still needs further research, regardless of the quality of the corpus or the construction of the semantic web. The MLP weighting algorithm can efficiently utilize the advantages of four various algorithms and further enhance the similarity calculation performance. Intuitively, by integrating multiple similarity algorithms, the MLP weighting algorithm can capture the correlated contextual, lexical, character, and syntactical information of various similarity algorithms, which can be further utilized to improve the precision of automatic RadLex coding.

## Discussion

The existing automatic coding algorithms of English radiology reports cannot be used directly for Chinese ones and the existing automatic coding algorithms of Chinese medical terms cannot be referred to due to the lack of Chinese radiology standard lexicon. Although automatic coding can be expected to improve gradually and handle the problem of radiology reports in the future, it is still facing challenges and worthwhile devoting much effort to automatic cross-language RadLex coding research. This study established a cross-language automatic RadLex coding system for Chinese radiology reports which proved to be successful in coding 3778 radiology-specific phrases from the structured radiology reports. This finding alleviated the burden of constructing the Chinese radiology standard lexicon to coding the radiology reports. The hybrid translation and MLP weighting similarity algorithm can be used not only for radiology reports but for other documents in the medical domain. Such topics are therefore worthy of research, application, and promotion.

Standardized coding of medical data is critical to the success of searching, sharing, and analyzing medical data. However, the standard English terminologies are unable to be translated into Chinese in a short time. The cross-language automatic coding method is of great use to convert the medical data to the standard terminology without much manpower for large-scale, multi-center medical data research [35]. The hybrid translation and MLP weighting similarity algorithm can not only be used in radiology but the general medical domain. It provides a reference for other similar cross-language automatic medical coding research.

Some limitations still exist in this work. High-quality translation of radiology phrases mainly relies on the coverage of the dictionaries. The Chinese–English radiology dictionary was developed as comprehensively as possible in this study, but there is still an extraordinary quantity of rare words beyond current dictionary coverage. It is also a possible direction for expanding the dictionary through crawler or other methods in the future. If the dictionary translation is comprehensive enough, the translation results will be more reliable without using Google translation.

Although the MLP weighting algorithm has achieved impressive performance in automatic RadLex coding, many efforts are still needed to improve the synonyms mapping performance. For example, there have been many studies of text similarity algorithms, the proposed MLP weighting algorithm just integrates four typical text similarity algorithms. As for future work, we intend to address this problem by integrating more text similarity algorithms and analyzing their influence on the automatic RadLex coding.

## Conclusion

Non-standardized Chinese radiology reports limit interoperability and usability. As the various usages of radiology reports become more and more common, the need for standardized radiology reports has increased. The benefits and challenges of adopting RadLex standardized codes have lately received great attention. An innovative and effective automatic cross-language RadLex coding approach combining hybrid translation and a dynamic Multilayer Perceptron weighting text similarity ensemble algorithm is proposed in the paper to solve the standardization of Chinese radiology reports and can be taken as a reference to automatically cross-language coding on other related domains.

## Data Availability

The datasets used during the current study are not publicly available due to the privacy of patients but are available from the corresponding author on reasonable request.

## Abbreviations

RadLex: Radiological lexicon
GNMT: Google Neural Machine Translation
MLP: Multilayer Perceptron
Wup: The Wu and Palmer metric
ICD-10: International Classification of Diseases, Tenth Revision
CBOW: Continuous bag-of-words model
E–C: English–Chinese
C–E: Chinese–English
UNK: Unknown words
LCS: Least Common Subsume
BP: Backpropagation
ReLU: Rectified linear units
CT: Computed tomography
IDCNN: Iterated Dilated Convolutional Neural Networks
CRF: Conditional random fields

## References

[1] Ganeshan D, Duong P-AT, Probyn L, Lenchik L, McArthur TA, Retrouvey M, Ghobadi EH, Desouches SL, Pastel D, Francis IR (2018). Structured reporting in radiology. Acad Radiol.

[2] Cramer JA, Eisenmenger LB, Pierson NS, Dhatt HS, Heilbrun ME (2014). Structured and templated reporting: an overview. Appl Radiol.

[3] Langlotz CP (2006). RadLex: a new method for indexing online educational materials. Radiol Soc North Am.

[4] Stanfill MH, Williams M, Fenton SH, Jenders RA, Hersh WR (2010). A systematic literature review of automated clinical coding and classification systems. J Am Med Inform Assoc.

[5] Pereira S, Névéol A, Massari P, Joubert M, Darmoni S (2006). Construction of a semi-automated ICD-10 coding help system to optimize medical and economic coding. Stud Health Technol Inform.

[6] Hohnloser JH, Pürner F, Kadlec P (1995). Coding medical concepts: a controlled experiment with a computerised coding tool. Int J Clin Monit Comput.

[7] Larson DB, Towbin AJ, Pryor RM, Donnelly LF (2013). Improving consistency in radiology reporting through the use of department-wide standardized structured reporting. Radiology.

[8] Farkas R, Szarvas G (2008). Automatic construction of rule-based ICD-9-CM coding systems. BMC Bioinform.

[9] Wheater E, Mair G, Sudlow C, Alex B, Grover C, Whiteley W (2019). A validated natural language processing algorithm for brain imaging phenotypes from radiology reports in UK electronic health records. BMC Med Inform Decis Mak.

[10] Baccianella S, Esuli A, Sebastiani F (2013). Variable-constraint classification and quantification of radiology reports under the ACR Index. Expert Syst Appl.

[11] Ning W, Yu M, Zhang R (2016). A hierarchical method to automatically encode Chinese diagnoses through semantic similarity estimation. BMC Med Inform Decis Mak.

[12] Chen Y, Lu H, Li L (2017). Automatic ICD-10 coding algorithm using an improved longest common subsequence based on semantic similarity. PLoS ONE.

[13] Sun Q, Zhang X (2018). An English–Chinese termbase of neological medical terms: a corpus-based project. Lexicography.

[14] Lei K, Si S, Wen D, Shen Y. An enhanced computational feature selection method for medical synonym identification via bilingualism and multi-corpus training. In: IEEE 2nd international conference on big data analysis (ICBDA), vol 2017. 2017; p. 909–14

[15] Gomaa WH, Fahmy AA (2013). A survey of text similarity approaches. Int J Comput Appl.

[16] Resnik P. Using information content to evaluate semantic similarity in a taxonomy. In: Proceedings of the 14th international joint conference on artificial intelligence, vol 1995. 1995; p. 448–53.

[17] Jiang Y, Li G, Feng J, Li W-S (2014). String similarity joins: an experimental evaluation. Proc VLDB Endow.

[18] Abdelrahman AM, Kayed A (2015). A survey on semantic similarity measures between concepts in health domain. Am J Comput Math.

[19] Garla VN, Brandt C (2012). Semantic similarity in the biomedical domain: an evaluation across knowledge sources. BMC Bioinform.

[20] Ristad ES, Yianilos PN (1998). Learning string-edit distance. IEEE Trans Pattern Anal Mach Intell.

[21] Niwattanakul S, Singthongchai J, Naenudorn E, Wanapu S. Using of Jaccard coefficient for keywords similarity. In: Proceedings of the international multiconference of engineers and computer scientists: 2013; 2013. p. 380–4.

[22] Le Q, Mikolov T. Distributed representations of sentences and documents. In: International conference on machine learning: 2014; 2014. p. 1188–96.

[23] Futia G, Vetro A, Melandri A, De Martin JC (2018). Training neural language models with SPARQL queries for semi-automatic semantic mapping. Procedia Comput Sci.

[24] Naili M, Chaibi AH, Ghezala HHB (2017). Comparative study of word embedding methods in topic segmentation. Procedia Comput Sci.

[25] Miller GA (1995). WordNet: a lexical database for English. Commun ACM.

[26] Wu Z, Palmer M. Verbs semantics and lexical selection. In: Proceedings of the 32nd annual meeting on Association for Computational Linguistics: 1994. Association for Computational Linguistics; 1994. p. 133–8.

[27] Guessoum D, Miraoui M, Tadj C. A modification of wu and palmer semantic similarity measure. In: UBICOMM 2016 tenth international conference on mobile ubiquitous computing, systems, services and technologies: 2016; 2016. p. 41–6.

[28] Xie S, Liu Y. Using corpus and knowledge-based similarity measure in maximum marginal relevance for meeting summarization. In: 2008 IEEE international conference on acoustics, speech and signal processing: 2008. IEEE; 2008. p. 4985–8.

[29] Mihalcea R, Corley C, Strapparava C (2006). Corpus-based and knowledge-based measures of text semantic similarity. AAAI.

[30] Zhao C, Wang Z (2018). GOGO: an improved algorithm to measure the semantic similarity between gene ontology terms. Sci Rep.

[31] Pal SK, Mitra S (1992). Multilayer perceptron, fuzzy sets, and classification. IEEE Trans Neural Netw.

[32] Castro W, Oblitas J, Santa-Cruz R, Avila-George H (2017). Multilayer perceptron architecture optimization using parallel computing techniques. PLoS ONE.

[33] Yan H, Jiang Y, Zheng J, Peng C, Li Q (2006). A multilayer perceptron-based medical decision support system for heart disease diagnosis. Expert Syst Appl.

[34] Yu B, Wei J: IDCNN-CRF-based domain named entity recognition method. In: 2020 IEEE 2nd international conference on civil aviation safety and information technology: 2020; 2020. p. 542–6.

[35] Li B, Li J, Jiang Y, Lan X (2019). Experience and reflection from China’s Xiangya medical big data project. J Biomed Inf.

